# Spleen tyrosine kinase: a novel pharmacological target for sepsis-induced cardiac dysfunction and multi-organ failure

**DOI:** 10.3389/fimmu.2024.1447901

**Published:** 2024-11-04

**Authors:** Chiara Verra, Maria Kerstin Paulmann, Jamila Wegener, Enrica Marzani, Gustavo Ferreira Alves, Massimo Collino, Sina Maren Coldewey, Christoph Thiemermann

**Affiliations:** ^1^ Centre for Translational Medicine and Therapeutics, William Harvey Research Institute, Barts & The London School of Medicine & Dentistry, Queen Mary University of London, London, United Kingdom; ^2^ Department of Anesthesiology and Intensive Care Medicine, Jena University Hospital, Jena, Germany; ^3^ Septomics Research Center, Jena University Hospital, Jena, Germany; ^4^ Department of Neurosciences “Rita Levi Montalcini”, University of Turin, Turin, Italy; ^5^ Pharmacology Unit, School of Pharmacy, University of Camerino, Camerino, Italy

**Keywords:** sepsis, SYK, cardiac dysfunction, MOF, cytokine storm

## Abstract

Sepsis is a systemic condition caused by a dysregulated host response to infection and often associated with excessive release of proinflammatory cytokines resulting in multi-organ failure (MOF), including cardiac dysfunction. Despite a number of effective supportive treatments (e.g. ventilation, dialysis), there are no specific interventions that prevent or reduce MOF in patients with sepsis. To identify possible intervention targets, we re-analyzed the publicly accessible Gene Expression Omnibus accession GSE131761 dataset, which revealed an increased expression of spleen tyrosine kinase (*SYK*) in the whole blood of septic patients compared to healthy volunteers. This result suggests a potential involvement of SYK in the pathophysiology of sepsis. Thus, we investigated the effects of the highly selective SYK inhibitor PRT062607 (15mg/kg; i.p.) on sepsis-induced cardiac dysfunction and MOF in a clinically-relevant, murine model of sepsis. PRT062607 or vehicle (saline) was administered to 10-weeks-old C57BL/6 mice at 1h after the onset of sepsis induced by cecal ligation and puncture (CLP). Antibiotics (imipenem/cilastatin; 2mg/kg; s.c.) and analgesic (buprenorphine; 0.05mg/kg; i.p.) were administered at 6h and 18h post-CLP. After 24h, cardiac function was assessed *in vivo* by echocardiography and, after termination of the experiments, serum and cardiac samples were collected to evaluate the effects of SYK inhibition on the systemic release of inflammatory mediators and the degree of organ injury and dysfunction. Our results show that treatment of CLP-mice with PRT062607 significantly reduces systolic and diastolic cardiac dysfunction, renal dysfunction and liver injury compared to CLP-mice treated with vehicle. In addition, the sepsis-induced systemic inflammation (measured as an increase in inflammatory cytokines and chemokines in the serum) and the cardiac activation of NF-kB (IKK) and the NLRP3 inflammasome were significantly reduced in CLP-mice treated with PRT062607. These results demonstrate, for the first time, that SYK inhibition 1h after the onset of sepsis reduces the systemic inflammation, cardiac dysfunction and MOF, suggesting a potential role of the activation of SYK in the pathophysiology of sepsis. Novel therapeutic strategies that inhibit SYK activity may be of benefit in patients with diseases associated with local or systemic inflammation including sepsis.

## 1 Introduction

Sepsis, a life-threatening organ dysfunction caused by an infection, has been acknowledged as one of the most common causes of morbidity and mortality among admissions to the intensive care unit (ICU) (1–3). Severe systemic infections with bacteria, fungi or viruses frequently trigger an uncontrolled host immune response leading to sepsis and approximately 11 million of deaths every year (4). Sepsis may progress to septic shock characterized by circulatory and metabolic abnormalities associated with an increase in mortality (5). Cardiac dysfunction, with systolic and diastolic impairments, is one of the major complications in sepsis aversively affecting the outcome (6–9). Most notably, the presence of sepsis-induced multi-organ failure (MOF) increases the mortality from 25% to 30% in patients with sepsis and from 35% to 40% in patients with septic shock, highlighting the urgent need for new and specific treatments for organ dysfunction (10). Indeed, the current management of sepsis in the ICU includes the administration of antimicrobials and supportive therapies (i.e. vasopressors, fluids resuscitation, ventilation, renal replacement therapy) (11, 12). Unfortunately, finding new effective treatments for sepsis proofs challenging due to the multiple factors and mechanisms underlying the pathophysiology of sepsis-induced organ damage and dysfunction (13, 14). Nevertheless, there is good evidence that the excessive local and systemic inflammation significantly contribute to the development of cardiac dysfunction and, generally, of MOF (15–18). In consequence, pharmacological treatments inhibiting the release of sepsis-triggered inflammatory mediators may prevent MOF.

Spleen tyrosine kinase (SYK) is a non-receptor tyrosine kinase involved in a wide range of biological functions, such as cell signaling relating to the formation of key mediators in the adaptive immune response and innate immunity. In addition, SYK-activation is also linked to a variety of non-immune functions such as bone resorption by osteoclasts and vascular development (19–21). SYK is expressed in cells of the hematopoietic lineage (e.g. B-cells, neutrophils, dendritic cells and macrophages), in which SYK is a key activator of the signaling pathways downstream of several immune receptors, such as B-cell receptors (BCRs), Fc receptors (FcR) and toll-like receptors (TLRs). Interestingly, SYK takes part in TLR4 signaling, and in the activation of the NF-κB pathway and the NLRP3 inflammasome, which are known to be crucial players in the pathophysiology of sepsis (22–28). Inhibition of SYK is beneficial in multiple autoimmune and inflammatory diseases, like rheumatoid arthritis, lupus and allergy (29–32). Given the vital contribution of SYK signaling in immunologically relevant pathways and in light of the recent discovery that SYK plays a role in acute lung injury (33), we hypothesized that SYK inhibition may represent a novel potential treatment for sepsis-induced cardiac dysfunction and multi-organ failure.

To get a better understanding of the potential role of SYK in sepsis, we have carried out a separate statistical analysis of the gene expression profile of *SYK* in healthy volunteers and patients with sepsis (see below), which showed an increase in the expression of *SYK* in patients diagnosed with septic shock after surgery. Based on this result and the role played by SYK in pro-inflammatory pathways involved in sepsis, we evaluated here, for the first time, the effects of a highly selective and potent SYK inhibitor (PRT062607 or P505-15) on both sepsis-induced cardiac dysfunction and MOF in a cecal ligation and puncture (CLP) murine model of sepsis (34–36). Moreover, we investigated the mechanisms underlying the observed effects of the SYK-inhibitor by measuring the effects of sepsis in the absence and presence of the pharmacological intervention on serum cytokines and chemokines and the activation of key pro-inflammatory pathways in the heart.

## 2 Materials and methods

### 2.1 Spleen tyrosine kinase gene expression in human septic shock patients

Original microarray data were obtained from Gene Expression Omnibus (GEO) under the accession GSE131761, published by Martínez-Paz et al. (37). The study has been conducted, amongst others, on n=81 post-operative patients diagnosed with septic shock 24h after surgery and on n=15 healthy volunteers of mixed age and gender. Martínez-Paz et al. recruited patients from the ICU at Hospital Clínico Universitario de Valladolid in Spain. Septic shock was diagnosed based on the Third International Consensus Definitions for Sepsis and Septic Shock (Sepsis-3 criteria). Additionally, fifteen healthy volunteers of similar age to the septic shock patients were included to normalize the gene expression data. RNA was extracted from whole blood of patients to obtain the total RNA, which was then hybridized with Agilent Whole Human Genome Oligo Microarray Kit following manufacturer’s instructions (37). Spleen tyrosine kinase (*SYK*) gene expression profile was analyzed in both septic shock and healthy human volunteers and results were then compared between the groups by unpaired *t*-test on GraphPad Prism 10 (GraphPad Software, Inc., La Jolla, CA, USA).

### 2.2 Animals and ethical statement

This study was conducted on 10-weeks-old male (n=15) and female (n=15) C57BL/6 mice (Charles River, UK), weighing 25-30g, kept under standard laboratory conditions. The animals were allowed to acclimatize to laboratory conditions for at least one week before any experimental procedure. Six mice were housed together in ventilated cages lined with absorbent bedding material. Tubes and chewing blocks were placed in all cages for environmental enrichment. All animals were subjected to 12-h light and dark cycles and the temperature was maintained at 19-23°C. All animals had free access to a standard chow diet and water. The cages were cleaned approximately every three days, with water being changed daily. Research staff inspected the animals each day for any signs of illness or abnormal behavior. The Animal Welfare Ethics Review Board of Queen Mary University of London (QMUL) approved all the *in vivo* experiments in accordance with the Home Office guidance on the Operation of Animals (Scientific Procedures Act 1986) published by Her Majesty’s Stationery Office and the Guide for the Care and Use of Laboratory Animals of the National Research Council. All research was conducted under U.K. Home Office project license number PP6747232. All *in vivo* experiments are reported in accordance to ARRIVE guidelines (38).

### 2.3 Experimental design

Ten-week-old male and female (n=30) C57BL/6 mice were randomly divided into 3 groups containing 5 males and 5 females each (n=10 total animals in each group): sham+vehicle (saline; i.p), CLP+vehicle (saline; i.p), CLP+SYKi (spleen tyrosine kinase inhibitor, 15mg/kg PRT062607 dissolved in saline; i.p.). The dose of 15mg/kg of PRT062607 was established based on previous studies of the pharmacokinetics of PRT062607 in animal models of inflammatory diseases. The dose of 15mg/kg of PRT062607 was effective in suppressing inflammation in rodent models of rheumatoid arthritis (36). CLP+vehicle and CLP+SYKi animals underwent CLP (more details below), whereas the sham+vehicle group was subjected to laparotomy without ligation and puncture of the cecum. Just before surgery, buprenorphine (0.05mg/kg; i.p.) was given as analgesic. PRT062607 or vehicle (saline) injection was given intraperitoneally at 1h after surgery. All groups of animals received antibiotics (imipenem/cilastatin; 2mg/kg dissolved in resuscitation fluid saline; s.c.) and buprenorphine (0.05mg/kg; i.p.) at 6h and 18h after CLP or sham surgery. At 24h after surgery and just before echocardiography, sepsis severity was evaluated using the murine sepsis score (MSS) and as previously described (39, 40): mild condition (MSS ≤ 1), moderate sepsis (1<MSS<3) and severe sepsis (MSS≥3). Echocardiography *in vivo* was then performed to assess the cardiac function of all groups of animals (see below for details). After echocardiography, mice were deeply sedated (4% isoflurane and 1L/min oxygen). Blood was obtained by cardiac puncture using a 18G needle and then centrifuged to obtain the serum. The serum was finally used for the information on the degree of MOF and the systemic release of cytokines and chemokines. Mice were then euthanized by removal of heart and lungs. Heart was collected, snap frozen in liquid nitrogen and kept stored at -80°C before performing further analysis.

### 2.4 Cecal ligation and puncture

Cecal ligation puncture (CLP), a surgical procedure considered to be the gold standard of *in vivo* sepsis models, was used to reproduce the clinical course of sepsis (41). Before the start of the procedure, mice received buprenorphine (0.05mg/kg, i.p.) as an analgesic. Anesthesia was induced by inhalation of 3% isoflurane and 1L/min oxygen, then maintained with 2% isoflurane and 1L/min oxygen via nosecone for all the duration of the surgery. Temperature of mice was monitored during the procedure and kept at 37°C with a homoeothermic mat. Fur on the mice abdomen was removed by Veet^®^ hair removal cream, and skin was then cleaned with saline. Then, 1.5cm midline incision was made to expose the cecum. The isolated cecum was ligated below the ileo-cecal valve and perforated at the top and at the bottom using an 18G needle to extrude a small amount of feces. Finally, the cecum was placed back to its anatomical position into the peritoneal cavity. Before suturing the abdomen, 5mL/kg of pre-warmed saline was administered into the opened cavity. One single dose of fluid resuscitation was given to mice after surgery using pre-warmed saline (10mL/kg; s.c.).

### 2.5 Assessment of cardiac function *in vivo*

Cardiac function was assessed by echocardiography *in vivo* using the Vevo-3100 imaging system (VisualSonics, Toronto, Ontario, Canada) after 24 hours from CLP or sham surgery. Anesthesia was induced with 3% isoflurane and 1L/min oxygen and maintained with 1.5% isoflurane and 1L/min oxygen via nosecone for the duration of the procedure. The heart rate and the internal body temperature were monitored over the procedure. Fur on the chest of the animals was removed and the skin was then cleaned. Pre-warmed echocardiography gel was then placed on the shaven chest of mice enabling to view the heart using the scan head probe. Systolic cardiac function was analyzed by measuring ejection fraction (EF%), fractional shortening (FS%), cardiac output (CO) and stoke volume (SV) related to the left ventricle. The apical four chamber plane was optimal for diastolic function assessment by obtaining the myocardial performance index (MPI) and the ratio between the velocity of the mitral valve (MV) “early” peak (E) and the “atrial” peak (A) (E/A). Blood flow was assessed by looking at the flow through the pulmonary artery and the aortic arch, measuring the value of pulmonary artery (PA), ascending (AscA) and descending aorta (DescA) peak velocity (Peak Vel) and velocity time integral (VTI).

### 2.6 Quantification of multi-organ failure degree in the serum

At the end of the echocardiography, serum samples were analyzed to assess the biomarkers of multi-organ injury and dysfunction in all mice. Approximately 1mL of blood was obtained by cardiac puncture (18G needle and non-heparinized syringe) and transferred into 1.3mL serum gel tubes (Sarstedt, Nürnbrecht, Germany). The serum was obtained by centrifuging the blood samples for 3min at 9000rpm. The aliquots of serum were then snap frozen in liquid nitrogen and stored at -80°C before sending them to an independent veterinary testing laboratory (Medical Research Council Harwell Institute, Oxford, England) to blindly quantify serum urea, creatinine, alanine aminotransferase (ALT), aspartate aminotransferase (AST), creatine kinase (CK) and lactate dehydrogenase (LDH).

### 2.7 Quantification of cytokines and chemokines in the serum

Cytokine and chemokine levels in the serum of mice were quantified with a Luminex xMAP Technology Bio-Plex™ 200 System (Bio-Rad, Kabelsketal, Germany). For this, serum was obtained as described above and samples processed using the Bio-Plex Pro™ Mouse Chemokine Panel 31-Plex assay kit (Bio-Rad, Kabelsketal, Germany) according to manufacturer’s specifications and as described previously (42). The following cytokines were analyzed as part of the panel: interleukin (IL)-1β, -2, -4, -6, -10, -16, I-309/CCL1, MCP-1/CCL2, MIP-1a/CCL3, MIP-1b/CCL4, RANTES/CCL5, MCP-3/CCL7, eotaxin/CCL11, MCP-5/CCL12, TARC/CCL17, MIP-3b/CCL19, MIP-3a/CCL20, MDC/CCL22, eotaxin2/CCL24, CTACK/CCL27, IFN-γ, TNF-α, as well as the chemokines fractalkine/CX3CL1, KC/CXCL1, ENA-78/CXCL5, IP-10/CXCL10, I-TAC/CXCL11, SDF-1a/CXCL12, BCA-1/CXCL13, SCYB16/CXCL16 and the growth factor GM-CSF. Quantification of the measured fluorescence data was done according to manufacturer’s (Bio-Plex Manger software v. 6.1.1; Bio-Rad, Kabelsketal, Germany) specification. The heatmap was generated using R version 4.3.2 (43).

### 2.8 Western blot analysis on cardiac tissue

Abundance of proteins of interest within heart samples (apical part) were evaluated by western blot analysis. Total protein extraction was performed accordingly: approximately 40mg of samples were weighed, then homogenized and centrifuged at 10.000g for 25min at 4°C. Protein quantity was assessed through bicinchonic acid (BCA, 23225 Pierce^®^ BCA Protein, Pierce Biotechnology Inc., Rockford, IL, USA) protein assay. Subsequently, proteins underwent electrophoretic separation on sodium dodecyl-sulphate polyacrylamide gel (SDS-PAGE) at 7%-8% polyacrylamide concentration (200 V/35 min) and were then transferred to nitrocellulose membranes (1215471 GVS North America, Inc., 0,45µm Nitrocellulose NC/MCE Membrane rolls) (100 V/70 min) using BIO-RAD mini-PROTEAN^®^ Tetra system. Membranes were blocked with 5% milk solution in TBS-Tween 0,1%, washed with TBS-Tween 0,1% and then incubated overnight at 4°C with the primary antibody diluted in BSA 1%. 1:1000 Rabbit anti-Ser^525/526^SYK (Cell signaling, #2710),1:1000 Rabbit anti-total SYK (Cell signaling, #13198), 1:1000 Rabbit anti-Ser^176/180^ IKKα/β (Cell Signaling, #2697S), 1:1000 rabbit anti-total IKKβ (Cell Signaling, #2370S), 1:1000 Rabbit anti-NLRP3 (Adipogen, #AG-20B-0014) were used as primary antibodies. On the following day, after washing the membranes, incubation was done with HRP-conjugated secondary antibodies anti-mouse (Cell signaling, #7076) and anti-rabbit (Cell Signaling #7074) diluted 1:7500 in 5% milk in TBS-Tween 0,1% at room temperature for one hour. The chemiluminescence signal was detected with the enhance chemiluminescence detection system (ECL, Bio-Rad Laboratories, Inc., Hercules, CA 94547, USA), bands were semiquantified with ImageLab (Version 6.1.0 BioRad Laboratories Software) and results are shown as phosphorylated/total protein ratio (SYK and IKK) or as total protein/loading control ratio (NLRP3).

### 2.9 Statistical analysis

All data in text and figures were expressed as mean ± standard error mean (SEM) of n observations, where n represents the number of patients/animals. All the statistical analyses were made on GraphPad Prism 10 (GraphPad Software, Inc., La Jolla, CA, USA). Outliers were identified by ROUT test (Q=1%). The normal distribution of the data was verified by Shapiro-Wilk test and the homogeneity of variances by Bartlett’s test. Statistical difference in the gene expression profile of human patients was analyzed by unpaired *t*-test. Statistical differences in the not normally distributed *in vivo* data were analyzed by Kruskal-Wallis test followed by Dunn’s multiple comparison test, whereas one-way ANOVA followed by a Bonferroni’s *post-hoc* test was applied to the normally distributed *in vivo* data. Pearson’s correlation with P-value based on two tailed test was used to determine correlation coefficients. All values with a P-value less than 0.05 were considered statistically significant.

## 3 Results

### 3.1 SYK gene expression increases in septic patients

**Figure 1:**
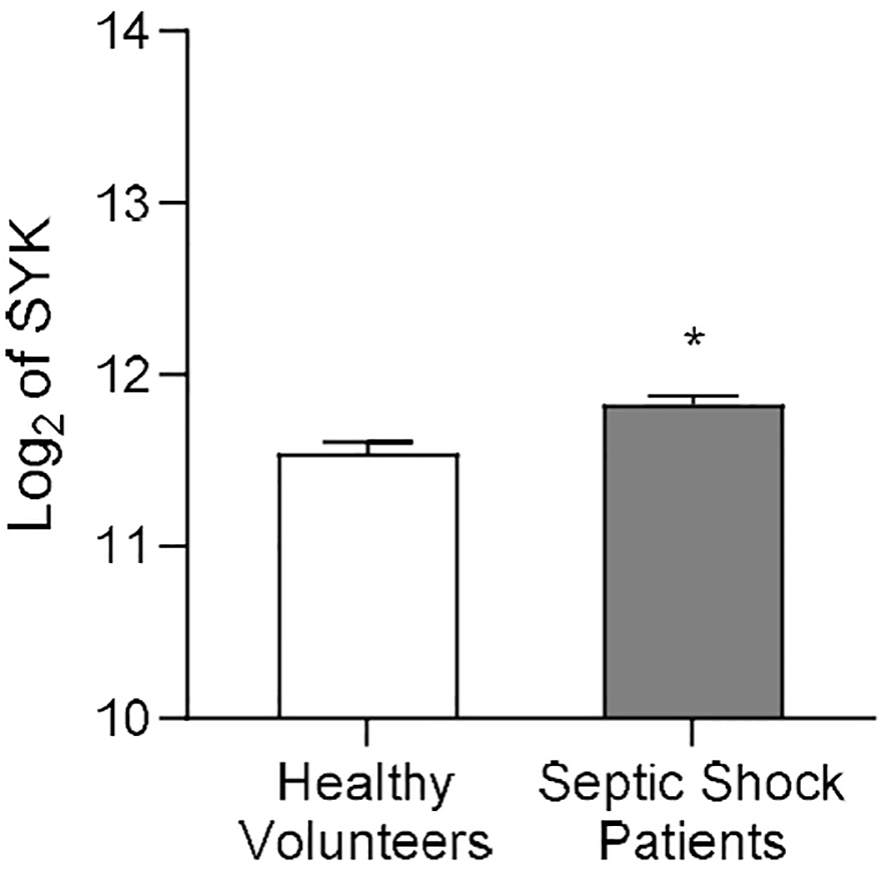
*SYK* gene expression increases in human patients diagnosed with septic shock after surgery. Data on *SYK* gene expression derived from the Gene Expression Omnibus under dataset accession number GSE131761, published by Martínez-Paz and colleagues (37). Whole blood of post-operative septic shock patients (Septic Shock Patients group, n=81) and from healthy volunteers (Healthy Volunteers group, n=15) of mixed age and gender was collected to extract RNA from each patient. Statistical difference between the groups (Healthy Volunteers and Septic Shock Patients) was analyzed by unpaired *t*-test. A value of *P<0.05 was considered to be statistically significant.

Martínez-Paz et al. collected whole blood of post-operative patients with confirmed septic shock and of healthy volunteers to evaluate gene expression patterns (37). We re-analyzed the clinical dataset for *SYK* gene expression, and the result showed that, when compared to healthy volunteers, *SYK* gene expression was significantly increased in patients diagnosed with septic shock 24h after surgery (P<0.05; **Figure 1**) compared to the healthy control group. This finding suggests a potential involvement of SYK in the pathophysiology of sepsis and septic shock.

### 3.2 The SYK-inhibitor PRT062607 reduces the systolic cardiac dysfunction induced by sepsis and ameliorates the clinical signs of morbidity

**Figure 2:**
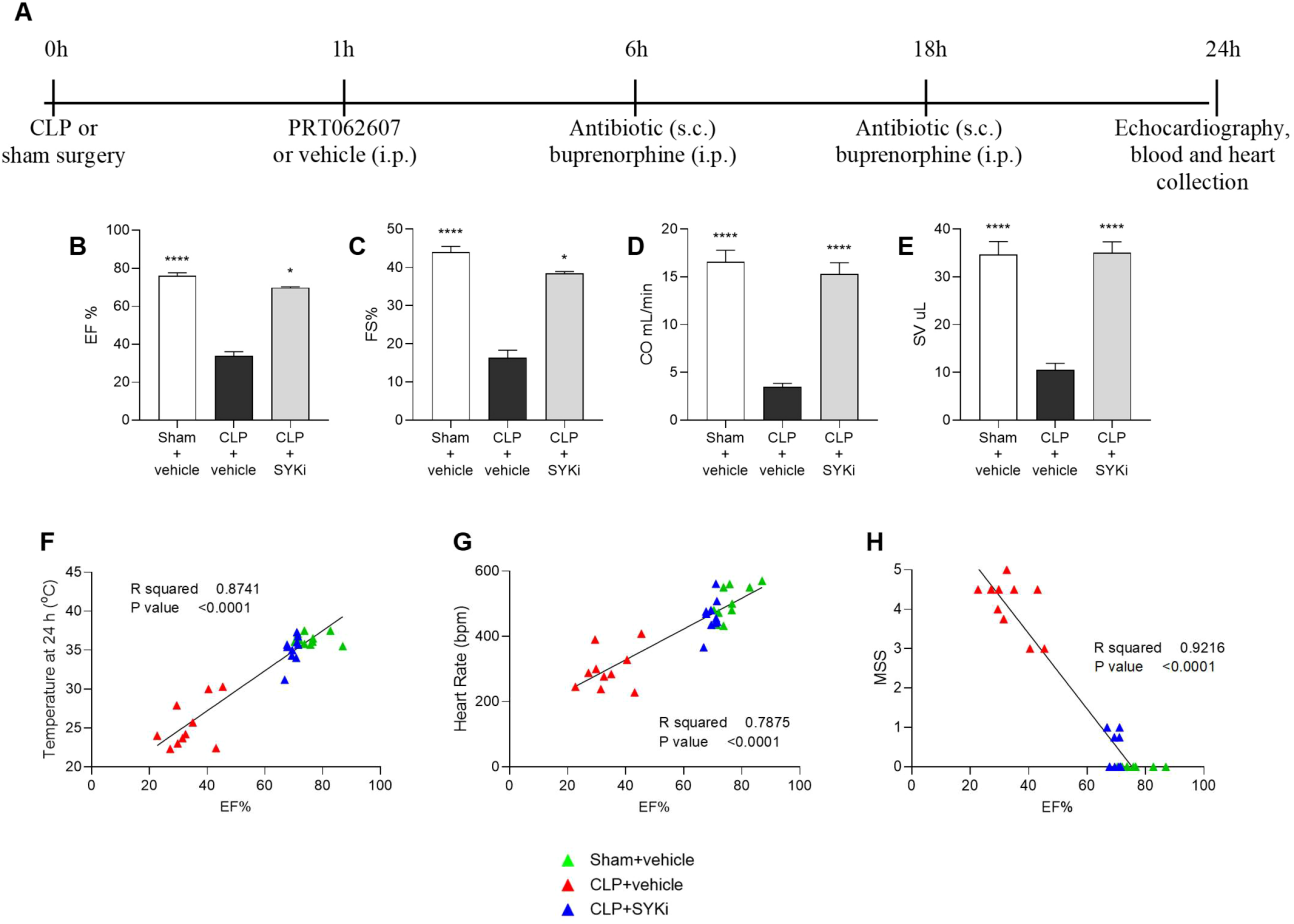
Effect of the SYK-inhibitor (SYKi) PRT062607 on CLP-induced systolic dysfunction, heart rate, temperature and morbidity (murine sepsis score). Mice were treated with PRT062607 (15mg/kg; i.p.) or vehicle 1h post-surgery (CLP or sham surgery). Cardiac function was assessed at 24h post-surgery by echocardiography *in vivo*. **(A)** Experimental design; **(B)** Ejection fraction (EF%); **(C)** Fractional shortening (FS%); **(D)** Cardiac output (CO); **(E)** Stroke volume (SV); **(F–H)** Correlation between EF% and temperature, heart rate and murine sepsis score (MSS) registered at 24h. The following groups (n=9-10 animals in each group) were studied: sham+vehicle, CLP+vehicle and CLP+SYKi. EF% and FS% data were analyzed by Kruskal-Wallis test followed by Dunn’s multiple comparison test, whereas CO and SV data were analyzed by one-way ANOVA, followed by Bonferroni’s *post-hoc* test. Data are expressed as mean ± SEM for n number of observations. *P<0.05 and ****P<0.0001 were considered to be statistically significant when compared to CLP+vehicle. Correlation coefficients were determined by Pearson’s correlation with P-values based on two-tailed tests.

Cardiac function was assessed in all mice at 24h after CLP or sham surgery (**Figure 2A**). The systolic function was analyzed by visualizing the left ventricle in M-mode. When compared to sham-operated mice (sham+vehicle), mice that underwent CLP and were treated with vehicle (CLP+vehicle) showed a reduction in EF%, FS%, CO and SV (P<0.001; **Figures 2B–E**), indicating left ventricular systolic dysfunction. In particular, EF% measures the percentage of blood ejected from the left ventricle per heartbeat, indicating heart pumping efficiency. FS% reflects the percentage reduction in left ventricular diameter during contraction, assessing contractility. SV is the volume of blood ejected per heartbeat, important for evaluating cardiac output. CO represents the total blood volume pumped per minute, calculated by multiplying SV and heart rate, and is essential for assessing overall cardiovascular health. CLP-mice treated with PRT062607 1h post-surgery (CLP+SKYi) showed an improved systolic left ventricular function compared to CLP+vehicle animals (P<0.05; **Figures 2B–E**), with cardiac parameters restored to values similar to sham-operated mice. Moreover, the EF% was positively correlated with the body temperature (R^2^ = 0.8741; **Figure 2F**) and the heart rate (R^2^ = 0.7875; **Figure 2G**), whereas it was negatively correlated with MSS (R^2^ = 0.9216; **Figure 2H**).

### 3.3 The SYK-inhibitor PRT062607 reduces the diastolic cardiac dysfunction induced by sepsis

**Figure 3:**
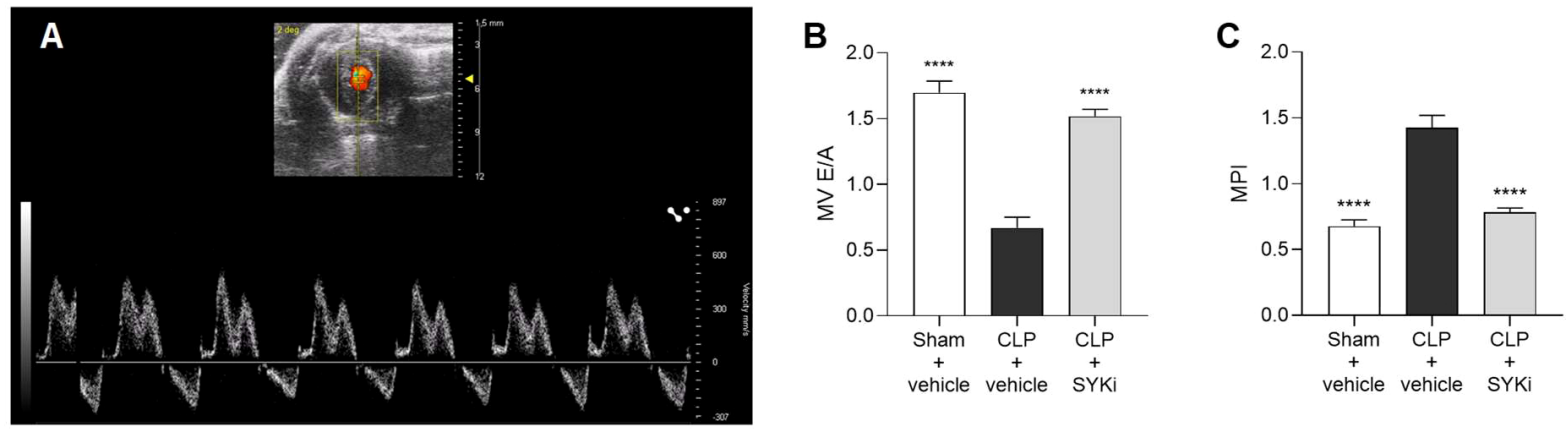
Effect of the SYK-inhibitor (SYKi) PRT062607 on CLP-induced diastolic dysfunction. Mice were treated with PRT062607 (15mg/kg; i.p.) or vehicle 1h post-surgery (CLP or sham surgery). Cardiac function was assessed at 24h post-surgery by echocardiography *in vivo*. **(A)** Mitral valve blood flow Pulsed Wave Doppler trace; **(B)** Mitral valve (MV) E/A ratio; **(C)** Myocardial Performance Index (MPI). The following groups (n=10 animals in each group) were studied: sham+vehicle, CLP+vehicle and CLP+SYKi. Data were analyzed by one-way ANOVA, followed by Bonferroni’s *post-hoc* test. Data are expressed as mean ± SEM for n number of observations. ****P<0.0001 was considered to be statistically significant when compared to CLP+vehicle.

The left ventricular diastolic function was assessed by measuring the blood flow through the mitral valve (**Figure 3A**). When compared to sham+vehicle group, CLP-mice treated with vehicle (CLP+vehicle) showed a drop in E/A ratio and an increase in MPI, both signs of diastolic function impairment (P<0.0001; **Figures 3B, C**). MPI provides a comprehensive assessment of overall cardiac function by evaluating both systolic and diastolic performance, offering insights into heart efficiency. MV E/A compares early and late diastolic flow velocities across the mitral valve to evaluate left ventricular filling pressures and diastolic function. In case of diastolic dysfunction, the ventricle struggles in creating the pressure gradient necessary to start the “early filling” (E wave) through the mitral valve, leading to a reduction in the passive flow during the E wave. In this scenario, more blood is actively pumped into the ventricle by the atrial contraction (A wave). As a result, the mitral valve trace of vehicle treated CLP-mice (CLP+vehicle) showed the A-wave larger than the E-wave, leading to E/A ≤ 1 (**Figure 3B**). Moreover, as a consequence of the extended duration of the isovolumic relaxation and contraction times, caused by the left ventricular diastolic and systolic dysfunction, the MPI incremented to more than 1 in CLP-animals treated with vehicle (**Figure 3C**). As can be deduced from the data reported here, 15mg/kg PRT062607 protected mice (CLP+SYKi) from sepsis-related diastolic dysfunction induced by CLP, improving both MV E/A and MPI when compared to CLP+vehicle group (P<0.0001; **Figures 3B, C**).

### 3.4 The SYK-inhibitor PRT062607 ameliorates the alterations in blood-flow through the pulmonary artery and the aorta caused by sepsis

**Figure 4:**
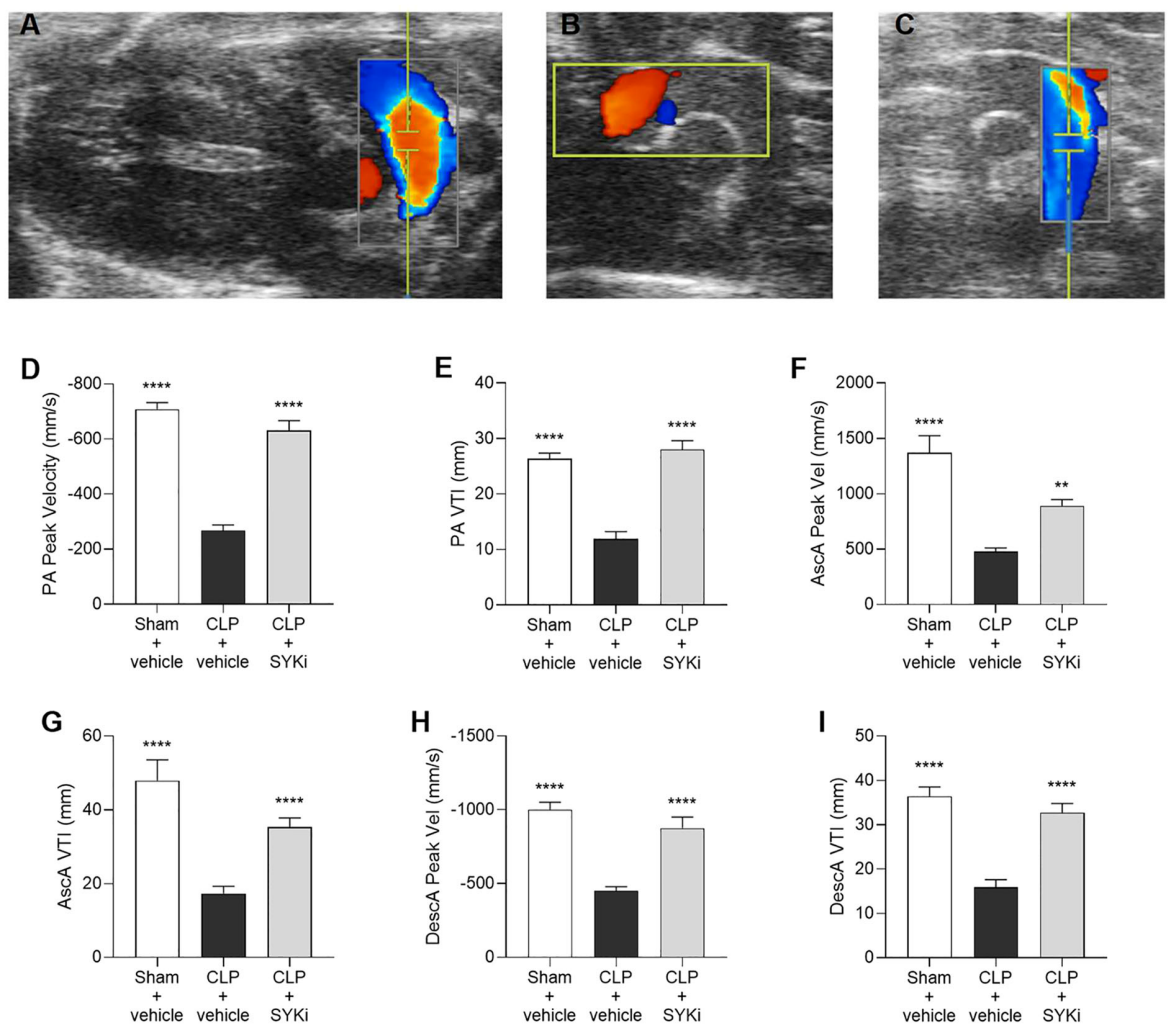
The SYK-inhibitor (SYKi) PRT062607 maintains the blood flow through the pulmonary artery and the aorta. Mice were treated with PRT062607 (15mg/kg; i.p.) or vehicle 1h post-surgery (CLP or sham surgery). Blood flow was assessed at 24h post-surgery by echocardiography in Color mode and Pulsed Wave Doppler mode *in vivo*. Echocardiography pictures in Color mode of: **(A)** pulmonary artery blood flow; **(B)** ascending aortic arch blood flow; **(C)** descending aortic arch blood flow. **(D, E)** Pulmonary artery (PA) peak velocity (Peak Vel) and velocity time integral (VTI); **(F, G)** ascending aorta (AscA) peak velocity (Peak Vel) and velocity time integral (VTI); **(H, I)** descending aorta (DescA) peak velocity (Peak Vel) and velocity time integral (VTI). The following groups (n=9-10 animals in each group) were studied: sham+vehicle, CLP+vehicle and CLP+SYKi. Ascending aorta peak velocity data were analyzed by Kruskal-Wallis test followed by Dunn’s multiple comparison test. All the other data in the figure were analyzed by one-way ANOVA, followed by Bonferroni’s *post-hoc* test. Data are expressed as mean ± SEM for n number of observations. **P<0.01 and ****P<0.0001 were considered to be statistically significant when compared to CLP+vehicle.

Giving the relationship between systolic and diastolic cardiac function and blood flow, the flow of blood through the pulmonary artery and the aortic arch was assessed by echocardiography in Color mode (**Figures 4A–C**) and then measured by Pulsed Wave Doppler mode. PA and aorta (ascending and descending tracts) peak velocity assess the highest blood flow speeds through these vessels, providing insights into right (PA) or left (aorta) ventricular function, with abnormal values suggesting ventricular dysfunction. VTI for both pulmonary artery and aorta (ascending and descending segments) measures the distance blood travels through the vessel in a single heartbeat providing insights into stroke volume, right (PA) or left (aorta) ventricular function, and cardiac output, with abnormal values pointing to potential cardiovascular issues. When compared to sham-operated mice, CLP-mice treated with vehicle (CLP+vehicle) showed a significant reduction in the pulmonary artery peak velocity and velocity time integral (P<0.0001; **Figures 4D, E**). Moreover, the blood flow through the aortic arch was also significantly reduced by sepsis in CLP+vehicle mice when compared to sham-operated mice (P<0.0001; **Figures 4F–I**). When compared to CLP-animals treated with vehicle (CLP+vehicle), animals that received 15mg/kg PRT062607 1h post-CLP showed a significant augmentation in pulmonary artery peak velocity and VTI (P<0.0001; **Figures 4D, E**) as well as an increase in flow through the aortic arch (specifically an increase in the ascending and descending aorta peak velocity and VTI; P<0.01; **Figures 4F–I**).

### 3.5 The SYK-inhibitor PRT062607 attenuates sepsis-induced multi-organ injury and dysfunction

**Figure 5:**
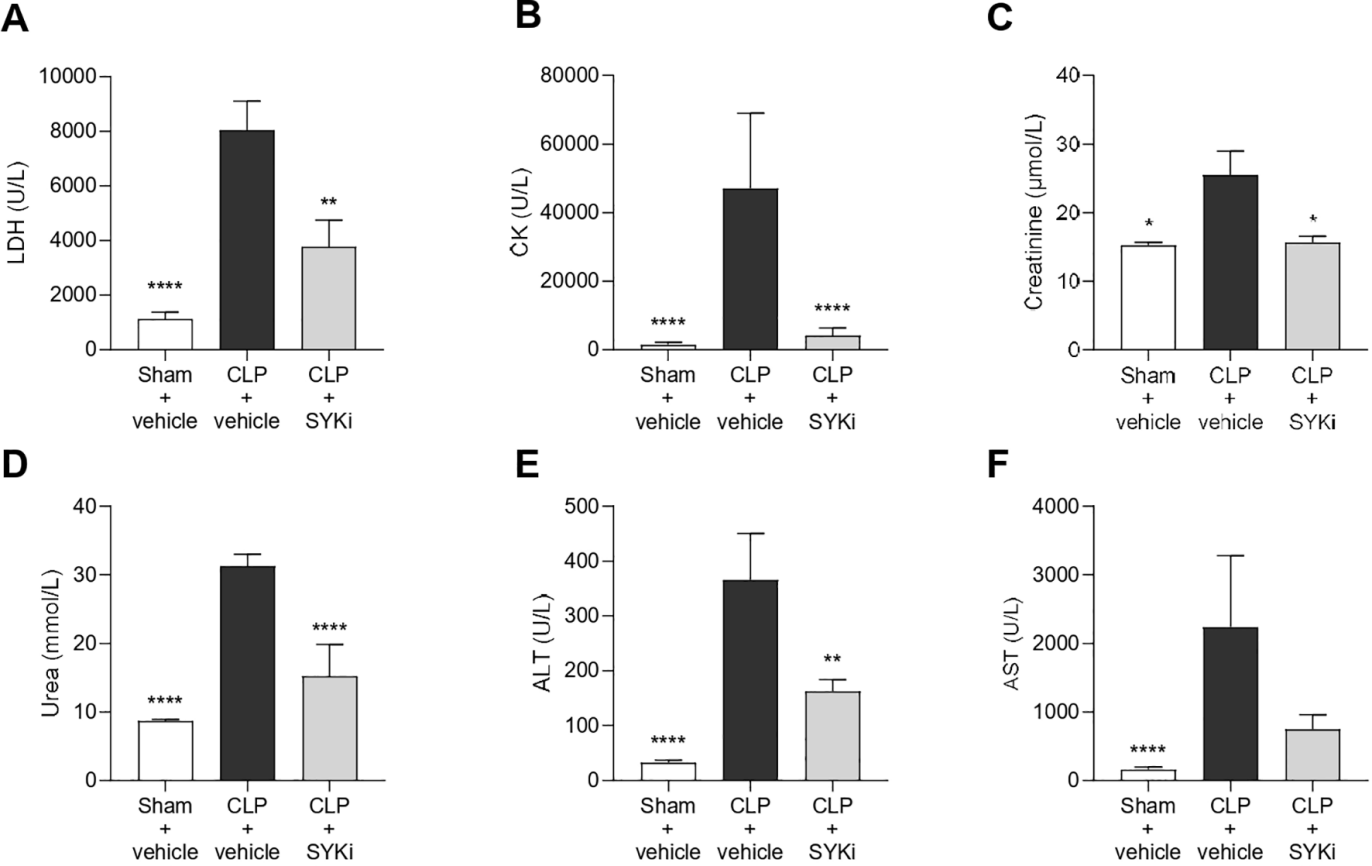
The SYK-inhibitor (SYKi) PRT062607 reduces the multi-organ dysfunction/injury caused by sepsis in CLP-mice. Mice were treated with PRT062607 (15mg/kg; i.p.) or vehicle 1h post-surgery (CLP or sham surgery). The levels of **(A)** lactate dehydrogenase (LDH), **(B)** creatine kinase (CK), **(C)** creatinine, **(D)** serum urea, **(E)** alanine aminotransferase (ALT) and **(F)** aspartate transaminase (AST) were measured in the serum at 24h after surgery. The following groups (n=8-10 animals in each group) were studied: sham+vehicle, CLP+vehicle and CLP+SYKi. Creatinine data were analyzed by Kruskal-Wallis test followed by Dunn’s multiple comparison test. Statistical differences in LDH, CK, serum urea, ALT and AST data were analyzed by one-way ANOVA, followed by Bonferroni’s *post-hoc* test. Data are expressed as mean ± SEM for n number of observations. *P<0.05, **P<0.01 and ****P<0.0001 were considered to be statistically significant when compared to CLP+vehicle.

Serum samples were collected to measure the degree of multi-organ injury and dysfunction in all groups of animals. In particular, biomarkers of hepatocellular injury (ALT and AST), renal dysfunction (urea and creatinine), cell-death (LDH) and skeletal muscle injury (CK) were measured in the serum. When compared to sham-operated animals, mice subjected to CLP and treated with vehicle showed increased levels of LDH, CK, creatinine, serum urea and both transaminase (ALT and AST), thus suggesting the development of multi-organ injury and dysfunction (P<0.05; **Figures 5A–F**). Treatment of CLP-mice with 15mg/kg PRT062607 at 1h after surgery significantly reduced the degree of cell death and skeletal muscle injury (P<0.01; **Figures 5A, B**). When compared to CLP+vehicle mice, CLP mice treated with PRT062607 showed a significant improvement in the renal function, with significantly reduced levels of creatinine and serum urea (P<0.05; **Figures 5C, D**). Regarding hepatocellular injury, serum level of ALT in CLP-mice that received PRT062607 was significantly reduced when compared to CLP-mice treated with vehicle (P<0.01; **Figure 5E**). However, the decrease in serum AST levels afforded by the PRT062607 treatment of CLP-mice was not significant but still evident when compared to CLP+vehicle group (**Figure 5F**).

### 3.6 PRT062607 potently inhibits SYK and reduces the activation of key inflammatory pathways in the heart of CLP-animals

**Figure 6:**
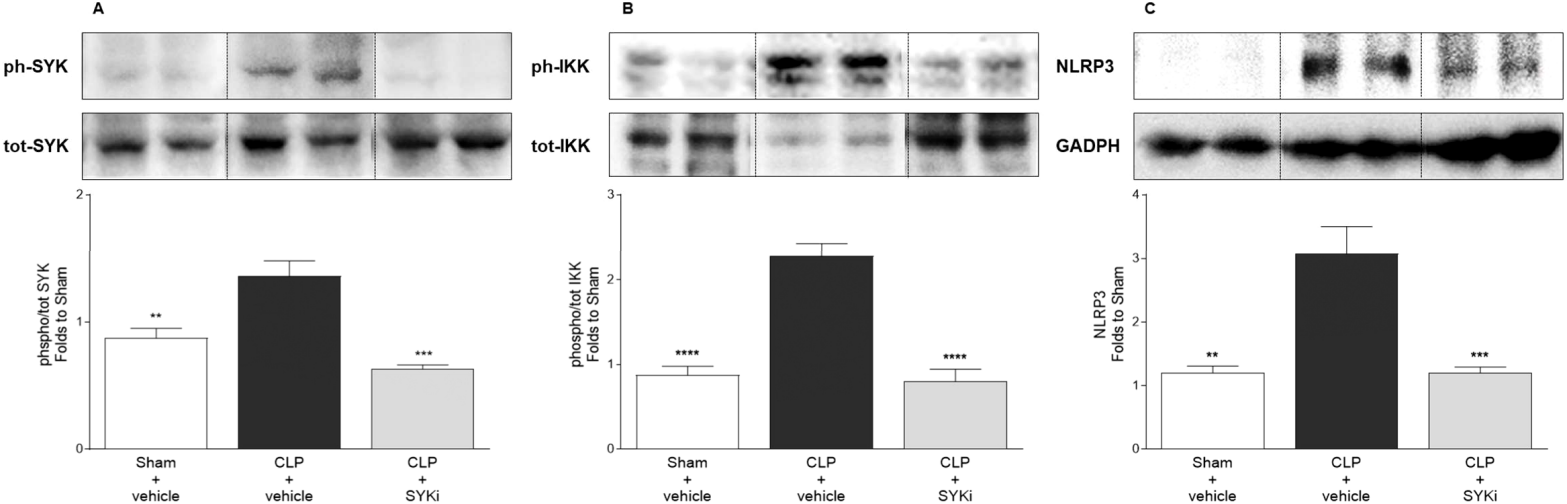
The SYK-inhibitor (SYKi) PRT062607 inhibits SYK and reduces IKK phosphorylation and NLRP3 expression in the heart of CLP-mice. Mice were treated with PRT062607 (15mg/kg; i.p.) or vehicle 1h post-surgery (CLP or sham surgery). Heart was collected at the end of the echocardiography (24h after surgery). Western blot analyses were conducted on cardiac tissue to determine **(A)** phosphorylation of SYK; **(B)** phosphorylation of IKK; **(C)** expression of NLRP3. Shown are representative western blot images. The following groups (n=4-5 animals in each group) were studied: sham+vehicle, CLP+vehicle and CLP+SYKi. Statistical differences were analyzed by one-way ANOVA, followed by Bonferroni’s *post-hoc* test. Data are expressed as mean ± SEM for n number of observations. **P<0.01, ***P<0.001 and ****P<0.0001 were considered to be statistically significant when compared to CLP+vehicle.

To get a better understanding of the mechanism by which 15mg/kg PRT062607 reduces the cardiac dysfunction caused by CLP-sepsis, we investigated the effects of PRT062607 on the cardiac activation of SYK (the target), the phosphorylation of IKK and the expression of NLRP3 (two key elements of inflammatory pathways activated in sepsis) by western blot analysis. When compared to sham-operated mice, the phosphorylation of SYK and, thus, the activation of the target, was significantly increased in the cardiac tissue of CLP-mice treated with vehicle (P<0.01; **Figure 6A**). Moreover, the activation of IKK (also known as IκB kinase) by phosphorylation and the expression of NLRP3 rose significantly in vehicle treated CLP-animals compared to sham-mice (P<0.01; **Figures 6B, C**). Administration of 15mg/kg of PRT062607 in CLP-mice significantly inhibited the activation of SYK in the cardiac tissue. Indeed, when compared to CLP-animals treated with vehicle, SYK phosphorylation was significantly abolished in CLP-animals that received PRT062607 (P<0.001; **Figure 6A**). Moreover, compared to CLP+vehicle mice, both IKK activation and NLRP3 expression were significantly reduced in the cardiac samples of CLP-mice treated with PRT062607 (P<0.001; **Figures 6B, C**).

### 3.7 The SYK-inhibitor PRT062607 attenuates the systemic inflammation caused by CLP in mice

**Figure 7:**
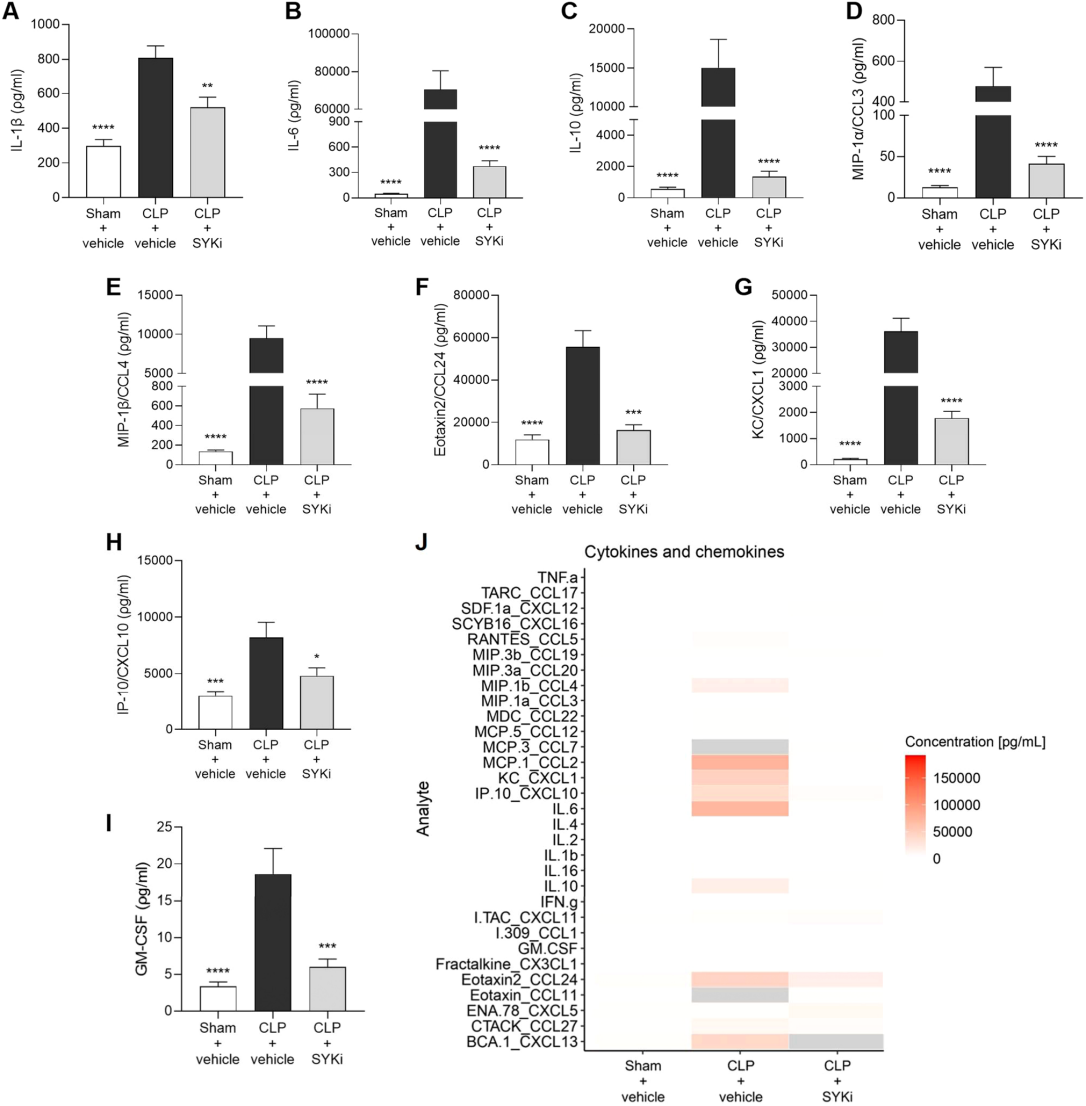
The SYK-inhibitor (SYKi) PRT062607 reduces the systemic inflammation caused by CLP-sepsis in mice. Mice were treated with PRT062607 (15mg/kg; i.p.) or vehicle 1h post-surgery (CLP or sham surgery). Serum was collected 24h after surgery to measure the concentration (ρg/ml) of 31 cytokines and chemokines. **(A)** IL-1β; **(B)** IL-6; **(C)** IL-10; **(D)** MIP-1α/CCL3; **(E)** MIP-1β/CCL4; **(F)** Eotaxin2/CCL24; **(G)** KC/CXCL1; **(H)** IP10/CXCL10; **(I)** GM-CSF; **(J)** Heat-map of the serum concentration (ρg/ml) of 31 cytokines and chemokines. The following groups (n=4-9 animals in each group) were studied: sham+vehicle, CLP+vehicle and CLP+SYKi. Data were analyzed by one-way ANOVA, followed by Bonferroni’s *post-hoc* test. Data are expressed as mean ± SEM for n number of observations. *P<0.05, **P<0.01, ***P<0.001 and ****P<0.0001 were considered to be statistically significant when compared to CLP+vehicle.

**Figure 8:**
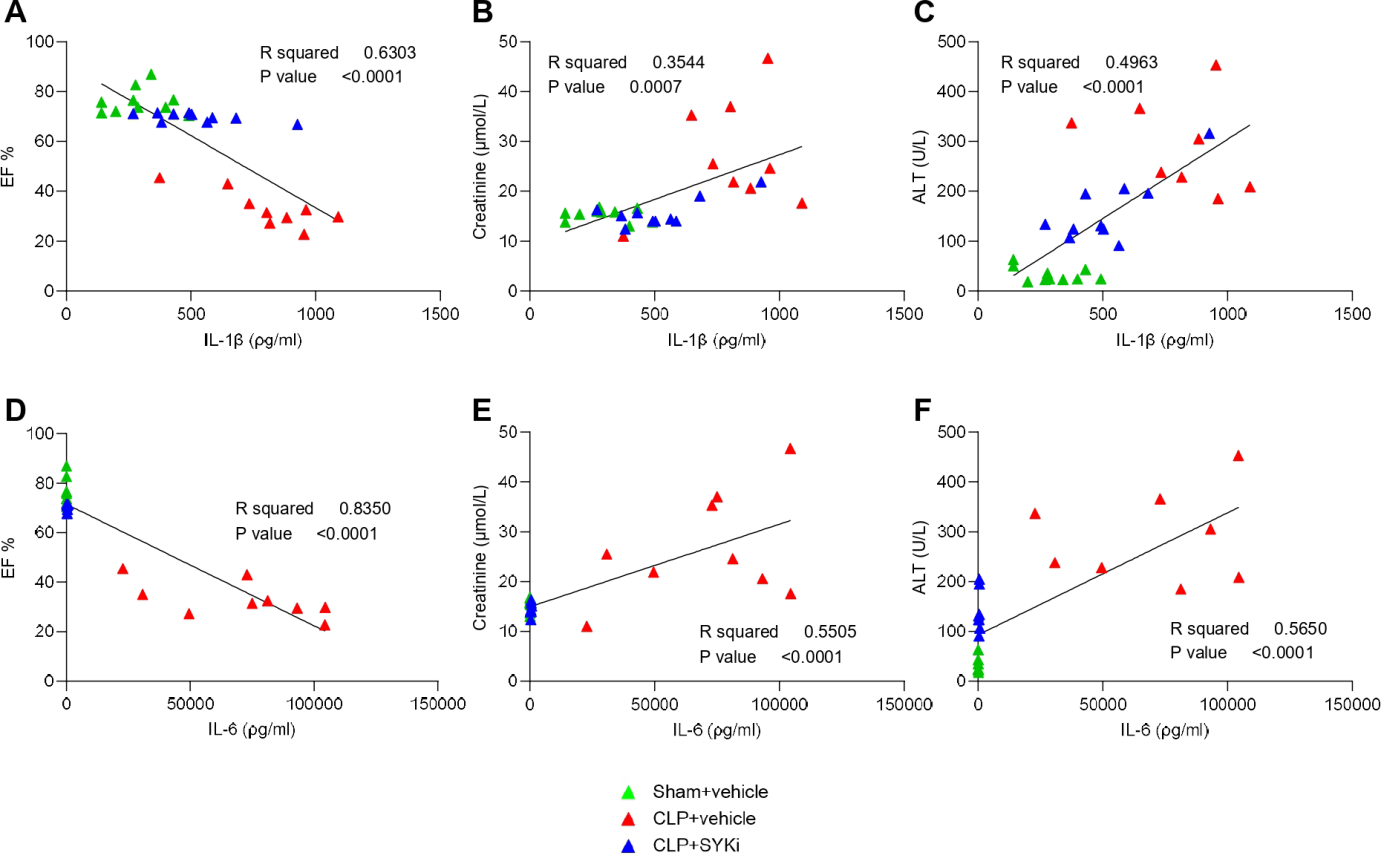
The SYK-inhibitor (SYKi) PRT062607 reduces cardiac and organ dysfunction by reducing CLP-induced systemic inflammation. Mice were treated with PRT062607 (15mg/kg; i.p.) or vehicle 1h post-surgery (CLP or sham surgery). Cardiac function was assessed at 24h post-surgery by echocardiography *in vivo* and serum was then collected to measure the concentration (ρg/ml) of 31 cytokines and chemokines and the level of the main organ function biomarkers. Correlation between IL-1β (ρg/ml) and **(A)** EF%, **(B)** Creatinine (µmol/L) and **(C)** ALT (U/L); **(D)** correlation between IL-6 (ρg/ml) and **(D)** EF%, **(E)** Creatinine (µmol/L) and **(F)** ALT (U/L). The following groups (n=10-8 animals in each group) were studied: sham+vehicle, CLP+vehicle and CLP+SYKi. Correlation coefficients were determined by Pearson’s correlation with P-values based on two-tailed tests.

To determine the effect of PRT062607 on CLP-induced systemic inflammation, the serum collected at 24h after surgery was analyzed by a multiplex array to quantify cytokines and chemokines. When compared to sham-operated mice (sham+vehicle), mice subjected to CLP-sepsis and treated with vehicle (CLP+vehicle) showed a significant increase in the serum levels of pro-inflammatory cytokines IL-1β, IL-6 and anti-inflammatory cytokine IL-10 (P<0.0001; **Figures 7A–C**). Moreover, CLP caused the significant rise in chemokines MIP-1α/CCL3, MIP-1β/CCL4, Eotaxin2/CCL24, KC/CXCL1, IP-10/CXCL10, and GM-CSF in CLP+vehicle mice, when compared to sham group (sham+vehicle) (P<0.001; **Figures 7D–I**). When compared to CLP+vehicle group, treatment of CLP-operated animals with 15mg/kg PRT062607 1h post-surgery significantly reduced the rise in cytokines and chemokines (P<0.05; **Figures 7A–I**). The alterations of further cytokines and chemokines can be consulted in the heat-map reported in **Figure 7J**. Moreover, IL-1β and IL-6 were negatively correlated with EF% (R^2^ = 0.6303, **Figure 8A**; R^2^ = 0.8350, **Figure 8D**), whereas they were positively correlated with the creatinine (R^2^ = 0.3544, **Figure 8B**; R^2^ = 0.5505, **Figure 8E**) and ALT (R^2^ = 0.4963, **Figure 8C**; R^2^ = 0.5650, **Figure 8F**). Thus, a high serum level of both IL-1β and IL-6 correlated with a low EF (cardiac dysfunction) and with high levels of both creatinine (renal dysfunction) and ALT (liver injury).

## 4 Discussion

Sepsis is one of the major public health problems characterized by a cascade of events, triggered by an infection, which culminates in multiple organ dysfunction and potentially death (5, 44, 45). Identifying a specific target for a potential new treatment for sepsis is challenging due to the multitude of molecular and metabolic alterations that occur in this syndrome. In the present study, we screened the publicly available GEO clinical dataset, that reports the gene expression of post-operative patients diagnosed with septic shock, to identify further genes that may have an important role (e.g. upregulated) in the pathophysiology of sepsis (37). Based on the results of our analysis, we identified SYK as a potential new target for sepsis-induced cardiac dysfunction and MOF. Indeed, when compared to healthy volunteers, the *SYK* gene is highly expressed in post-operative patients diagnosed with septic shock (**Figure 1**), and this may contribute to the pathophysiology of sepsis and, hence, cardiac dysfunction and MOF.

In order to address this hypothesis, we subsequently investigated the effects of a novel, highly selective SYK inhibitor, PRT062607 (or P505-15), on myocardial (dys)function and MOF in a clinically relevant, murine model of sepsis (36). To date, Fostamatinib (R788) is the only SYK inhibitor approved by both the EMA and FDA for human use (46, 47). Fostamatinib, the pro-drug of the active metabolite R406, is licensed for the treatment of patients with chronic immune thrombocytopenia (ITP) that do not respond to other treatments, but it is under evaluation for the treatment of other autoimmune diseases (30, 48–51). Fostamatinib has many off-target effects relating to the inhibition of other kinases (like FMS-related tyrosine kinase 3, Lck, Janus kinase 1 and 3, and c-kit). The small molecule PRT062607 inhibits SYK with an anti-SYK activity that is 80-fold greater than its affinity for other kinases, indicating that the molecule may have a better therapeutic window and, hence, may represent a good candidate to investigate the role of SYK in sepsis and other clinical conditions with an activated immune system (35, 36, 52–54).

In this study, sepsis was induced in mice by the CLP surgical procedure, which is regarded as the gold standard of sepsis animal models. Unlike the injection of LPS (or other wall fragments of bacteria), CLP causes a polymicrobial infection, recreating the progressive release of inflammatory mediators and, hence, the progression of the syndrome also seen in patients with sepsis. Most notably, CLP is able to reproduce the dynamic changes in cardiovascular function and the MOF observed in patients with sepsis (55–57). In the current study, we show that CLP-sepsis resulted in both systolic and diastolic cardiac dysfunctions. In particular, the main parameters of systolic contractility, EF%, FS%, CO and SV, which define the strength and contraction capacity of the left ventricle, were significantly reduced in CLP-mice treated with vehicle (**Figures 2B–E**) (58–60). Moreover, CLP-sepsis severely affected the relaxation capacity of the left ventricle, thus, in addition to reducing the MV E/A ratio, CLP increased the MPI in CLP-mice that received the vehicle (**Figures 3B, C**) (61–63). As a result of the cardiac dysfunction, the blood flow through the pulmonary artery and aorta was reduced in CLP-mice treated with vehicle. In CLP-mice, VTI and peak velocity related to aorta’s ascending and descending tract were both reduced reflecting the development of a systolic, cardiac dysfunction. Data on pulmonary artery blood flow allowed us to get an insight into the function of the right ventricle of vehicle-treated CLP-mice (**Figures 4F–I**). Indeed, pulmonary artery VTI and peak velocity were also reduced in CLP-mice, indicating that CLP-sepsis was also associated with a significant dysfunction of the right ventricle reducing blood flow through the pulmonary circulation (**Figures 4D, E**).

Here we show, for the first time, that the SYK inhibitor PRT062607 (15mg/kg administered at 1h after surgery) protects CLP-mice from sepsis-induced systolic and diastolic cardiac dysfunction of the right and left ventricle. Indeed, the set of cardiac parameters related to left ventricular systolic and diastolic function and the data on the aortic arch blood flow of CLP-mice treated with PRT062607 were, within 24h, nearly restored to normal levels (**Figures 2B–E**; **Figures 3B, C**; **Figures 4F–I**). Administration of PRT062607 also ameliorated sepsis-induced alterations in right ventricular function, as demonstrated by the significant increase in the pulmonary artery VTI and peak velocity (**Figures 4D, E**). The preservation of the cardiac function afforded by the SYK inhibitor PRT062607 was also associated with a protective effect against the development of hypothermia and bradycardia, both of which are common clinical signs of septic shock in patients with sepsis (**Figures 2F, G**) (64–67). Moreover, the severity of sepsis, assessed by the MSS score, which incorporates piloerection, tremors, respiratory distress, and periorbital exudates of the animals, was either less severe or not detectable in CLP-animals that received PRT062607, when compared to CLP-animals treated with vehicle. Notably, the MSS remained below 2 in CLP-mice treated with PRT062607, providing additional confirmation of reduction by SYK inhibition of the key clinical signs of sepsis (**Figure 2H**).

CLP-mice treated with vehicle developed sepsis with a decrease in the ability of myocardium to contract and, as a result, to satisfy the nutrient requirements of organs by providing a sufficient circulation of blood. The circulatory failure, together with the sepsis-related excessive inflammation, described here by the rise in circulating pro-inflammatory cytokines (**Figure 7**), contributes to the development of multi-organ injury and dysfunction in vehicle-treated CLP-mice (68). The rising levels of LDH confirmed the multi-organ damage/dysfunction in CLP-mice treated with vehicle (**Figure 5A**). Human septic patients frequently show high levels of LDH and an increase in this cytoplasmic enzyme in the serum has been linked to the severity of tissues damage, as it is released in the blood in case of cellular injury (69, 70). Multi-organ failure is a common complication in septic patients that further worsen the overall clinical picture. Apart from the heart, others most affected organs in sepsis are kidney and liver (71). CLP-sepsis caused renal dysfunction and hepatocellular injury in CLP-mice treated with vehicle, measured as pronounced increases of serum creatinine, serum urea and the hepatic transaminases (ALT and AST) (**Figures 5C–F**) (72–75). Moreover, vehicle treated CLP-mice showed an increase in CK, the main marker of skeletal muscle injury or breakdown (**Figure 5B**), which has also been reported in septic patients (76, 77). The analysis of these parameters (LDH, CK, creatinine, serum urea, ALT and AST) in CLP-mice treated with PRT062607 affirmed that the SYK inhibitor also reduced the renal dysfunction and hepatocellular injury caused by sepsis (**Figures 5A-F**).

What, then, is the mechanism(s) by which the SYK-inhibitor PRT062607 reduces the cardiac dysfunction and MOF in murine sepsis? In order to gain a better insight into the potential mechanism of action of PRT062607, we investigated the effects of sepsis (in the absence or presence of drug treatment) on a) the activation of the target, SYK, and b) pivotal pro-inflammatory pathways activated during sepsis. Given the key role played by the cardiac and circulatory (dys)function in the development of organ failure and, hence, in the outcome of septic patients, we focused our attention on the cardiac tissue of CLP-animals in order to address the mechanism of action of the beneficial effects of the SYK-inhibitor PRT062607 observed in murine sepsis (78–80). There is strong evidence that the NF-κB (nuclear factor-κB) pathway and the NLRP3 (NOD leucine-rich-repeat and pyrin domain–containing protein 3) inflammasome play a pivotal role in the pathophysiology of septic cardiomyopathy (and MOF), and the inhibition of both pathways contributes to the protective effects of a number of experimental interventions in murine sepsis (81–86). Therefore, we studied the cardiac activation of the NF-κB pathway by measuring the degree of IKK phosphorylation, and we quantified the cardiac levels of the NLRP3 protein. CLP-sepsis resulted in a significant increase in the phosphorylation and, hence, activation of SYK in cardiac tissue, which was inhibited by the administration of PRT062607 (**Figure 6A**). Interestingly, the inhibition of SYK phosphorylation afforded by PRT062607 was also associated with a reduced activation of IKK and reduced levels of NLRP3 in the heart of CLP-animals treated with PRT062607 (**Figures 6B, C**).

One explanation for the SYK inhibitor-dependent reduced activation of IKK may the fact that SYK acts as an upstream regulator of the NF-κB pathway in LPS-primed macrophages. Under inflammatory conditions, SYK has been reported to participate in TLR4-mediated responses by interacting and cooperating with the myeloid differentiation primary response gene 88 (MyD88) and other TLR4-associated intracellular adaptor molecules (28). In particular, after LPS-mediated stimulation of TLR4, SYK regulates the activity of the serine/threonine kinase IRAK1 (IL-1R-associated kinase 1), which then interacts with TRAF6 (TNF receptor-associated factor 6) driving IKK phosphorylation and, finally, NF-κB translocation to the nucleus, where it regulates the expression of various cytokines (87–89). In line with this, we show here that the CLP-induced activation of SYK was accompanied by a pronounced, systemic release of pro-inflammatory mediators, which are dependent of the activation of NF-κB. Specifically, we report here that CLP-sepsis caused a significant increase in IL-1β, IL-6, CCL3, CLL4, CXCL1, CXCL10 and GM-CSF, all of which contribute to sepsis-induced cytokine storm and, hence, to cardiac and organ dysfunction (17, 90–95). Most notably, the systemic inflammation, measured as the rise in cytokines and chemokines in the serum, and the cardiac activation of IKK and, hence, the NF-κB pathway, were reduced by the administration of the SYK inhibitor PRT062607 in CLP-animals (**Figure 6B**; **Figure 7**). Interestingly, we also report that a high serum level of IL-1β and IL-6 correlates with a low EF (cardiac dysfunction) and with high levels of both creatinine (renal dysfunction) and ALT (liver injury). Taken together, our findings show that PRT062607 is a potent small molecule, which inhibits the activation of the SYK-NF-κB axis, which in turn is a key player in the inflammatory response during sepsis and, hence, in sepsis-induced cardiac and organ dysfunction.

The NF-κB signaling pathway also plays a role in the formation of the NLRP3 inflammasome, another key element of innate immunity against infections. Specifically, NF-κB is a direct regulator of the transcriptional expression of NLRP3 and pro-IL-1β, two components necessary for inflammasome formation and activation, in response to various stimuli, including the activation of TLR4 by bacterial LPS. Indeed, the promoter region of both NLRP3 gene and pro-IL-1β gene contains NF-κB biding sites, representing two targets for NF-κB (96–98). Once formed, the NLRP3 inflammasome activates caspase-1, which subsequently mediates the cleavage of pro-IL-1β and pro-IL-18 and the secretion of their bioactive forms, important players in the systemic inflammation and cardiac dysfunction (and MOF) during sepsis (81–83, 99, 100). Our results show that CLP-sepsis resulted in a rise in the cardiac phosphorylation of IKK and, hence, activation of NF-κB pathway, which was associated with an increased expression of NLRP3 in the heart and augmented systemic release of the cardio-suppressive IL-1β (78, 90). Most notably, both the increase in cardiac NLRP3 and the serum levels of IL-1β were reduced by the inhibition of SYK with PRT062607, and this was accompanied by a reduction in IKK activation and an improved cardiac function (**Figures 6B, C**; **Figure 7A**). These findings suggest that the observed beneficial effects on the cardiac function caused by the administration of PRT062607 in CLP-animals may be the result of the reduced NLRP3 inflammasome assembly and activation, potentially due to the lower expression of NLRP3 and pro-IL-1β triggered by NF-κB. Thus, these observations further confirm the crucial role of SYK-NF-κB axis in sepsis-induced cardiac dysfunction and point out a potential mechanism of action underlying the protective effect provided by the SYK inhibitor PRT062607 against the development of sepsis-induced cardiac and multi-organ dysfunction.

SYK itself has also been linked to the formation and activation of the NLRP3 inflammasome caused by pathogens. Specifically, SYK causes ASC phosphorylation and oligomerization, which in turn drives the assembly of the NLRP3 inflammasome (25, 26, 101–103). In this study we show that CLP-sepsis increased the serum levels of the cytokine IL-1β, which is secondary (at least in part) to the activation of NLRP3 inflammasome during sepsis. Indeed, we and others have reported that CLP-sepsis causes both the activation of NLRP3 inflammasome and proteolytic cleavage of pro-caspase-1 to its active form caspase-1, finally leading to IL-1β maturation and release (40, 102, 104, 105). Importantly, we report here that the increase in serum IL-1β in CLP-animals treated with vehicle was associated with cardiac dysfunction and MOF. Most notably, the administration of the SYK inhibitor PRT062607 caused a reduction in the cytokines storm, including serum IL-1β, and an amelioration in the cardiac and other organs function. Thus, the reduction in the systemic release of IL-1β sustained by the administration of PRT062607 in CLP-animals may also be the result of the interplay between SYK and NLRP3 inflammasome (**Figure 7A**).

### 4.1 Limitations of the study

The UK Home Office license, under which this research was conducted, does not allow us to perform mortality studies. Thus, we were unable to investigate the effects of 15mg/kg PRT062607 administered 1h post-surgery on the mortality rate in mice with sepsis. In this study, we used surrogate markers of outcome by evaluation of physiological parameters, such as body temperature, heart rate and MSS, and the assessment of the cardiac function and the degree of MOF.

## 5 Conclusion

This study reports for the first time that inhibition of SYK-activation reduces cardiac dysfunction and, hence, ameliorates renal dysfunction and hepatocellular injury in a clinically relevant murine model of sepsis. Our results support the view that interventions which inhibit the activation of SYK are of potential therapeutic benefit in conditions associated with systemic (or local) inflammation including sepsis.

## Data Availability

The raw data supporting the conclusions of this article will be made available by the authors, without undue reservation.
